# A case of carcinoma of the papilla of Vater in a young man after subtotal colectomy for familial adenomatous polyposis

**DOI:** 10.1186/s12957-016-0806-8

**Published:** 2016-02-24

**Authors:** Shuji Komori, Masahiko Kawai, Toyoo Nitta, Yusuke Murase, Keita Matsumoto, Chika Shinoda, Masashi Kuno, Yuki Sasaguri, Masahiro Fukada, Yoshimi Asano, Shigeru Kiyama, Chihiro Tanaka, Yasuko Nagao, Narutoshi Nagao, Katsuyuki Kunieda

**Affiliations:** Department of Surgery, Gifu Prefectural General Medical Center, 4-6-1 Noisshiki, Gifu, 500-8717 Japan

**Keywords:** Familial adenomatous polyposis, Duodenal and ampullary cancer, Modified Imanaga reconstruction, Pancreaticoduodenectomy

## Abstract

**Background:**

Carcinoma and adenoma of the duodenum, including the papilla of Vater, are problematic diseases in patients with familial adenomatous polyposis (FAP).

**Case presentation:**

A 36-year-old man underwent a periodic medical examination for early colon cancer originating from FAP for which laparoscopic-assisted subtotal colectomy with a J-shaped ileal pouch-rectal anastomosis was performed 3 years earlier. A tumor was detected at the papilla of Vater along with elevation of total bilirubin and hepatobiliary enzymes. Although cytology did not determine the tumor to be an adenocarcinoma, we suspected adenocarcinoma due to its hypervascularity shown by contrast-enhanced computed tomography. Pylorus-preserving pancreaticoduodenectomy with modified Imanaga reconstruction and regional lymph node dissection (D2) was performed. The pathological study showed that the tumor was a papillary and moderately differentiated tubular adenocarcinoma. The patient is currently in good health without recurrence, weight loss, or severe diarrhea at 12 months after surgery.

**Conclusions:**

Awareness of biliary-pancreatic symptoms and periodic gastroduodenoscopy might contribute both to the early detection of duodenal or periampullary polyps and cancer and to the radical treatment of FAP. Modified Imanaga reconstruction has the potential to become one of the more effective procedures for providing good quality of life to FAP patients with duodenal or periampullary cancer.

## Background

Carcinoma and adenoma of the duodenum, including the papilla of Vater, are problematic diseases in patients with familial adenomatous polyposis (FAP), especially as duodenal polyps were detected in 65 % of FAP patients with a median age of 38 years [1]. Duodenal adenoma changes to adenocarcinoma via the adenoma-carcinoma sequence, but the incidence rate is only approximately 5 % of all polyps [2–4]. The establishment of endoscopic management and treatment of patients with FAP based on the Spigelman scoring system contributes to surveillance alone or to endoscopic mini-invasive treatment of stage 0–III patients, although even now, almost all patients with stage IV disease and cancer require short-term surveillance or endoscopic treatment or surgery [5].

For stage IV patients, several treatments, including endoscopic mucosal resection, ampullectomy, and pancreas-preserving duodenectomy (PpD)/pylorus-preserving pancreaticoduodenectomy (PpPD), have been discussed and recommended [5–7]; however, the patients managed with endoscopic resection of adenomas continue to be at substantial risk of developing recurrent adenomas [7]. For patients with duodenal or ampullary cancer, PpD/PpPD is selected in most cases [6, 8, 9].

We report a case of the onset of cancer of the papilla of Vater in a young man after subtotal colectomy for FAP. The available diagnostic and therapeutic strategies for surgical treatment are also discussed in light of the experience with the present case and in reference to previously reported cases.

## Case presentation

**Fig. 1:**
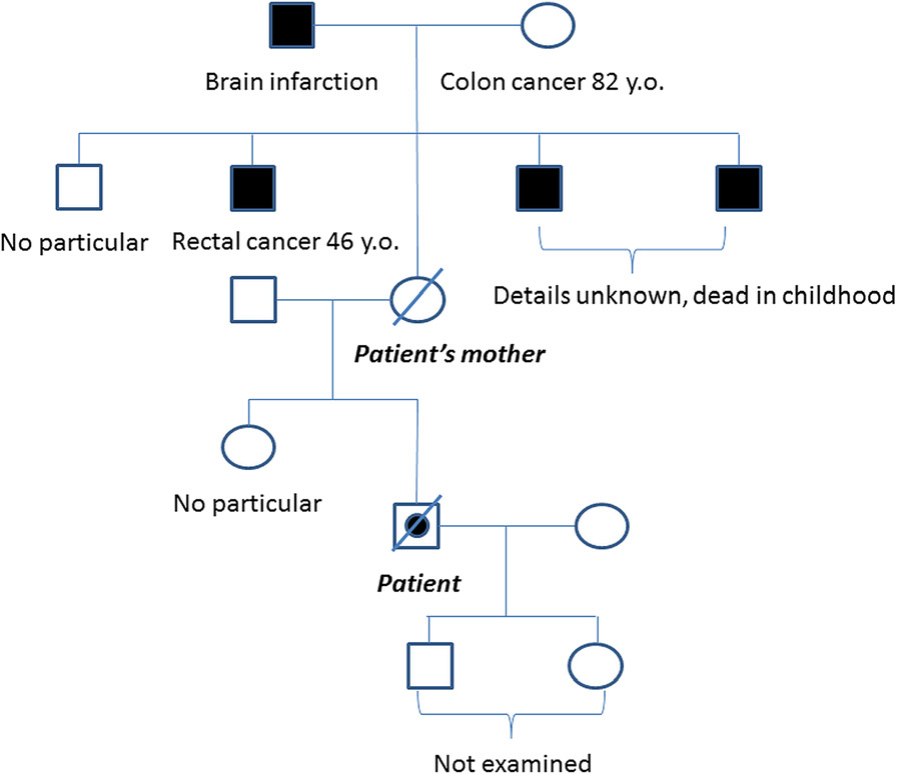
The patient’s family tree

A 36-year-old man underwent a periodic medical examination for early colon cancer originating from classical FAP (adenomatous polyposis coli (*APC*) gene in 5q21 analysis, codon 1–1800: exon 15, codon 795, 1 bp deletion of C: CTC→CT) for which laparoscopic-assisted subtotal colectomy with a J-shaped ileal pouch-rectal anastomosis was performed 3 years earlier. His mother also has FAP and the same gene mutation as with his subtype and gene and underwent the same operation 13 years earlier and resection of an intra-abdominal desmoid tumor 10 years earlier (Fig. 1).

**Fig. 2:**
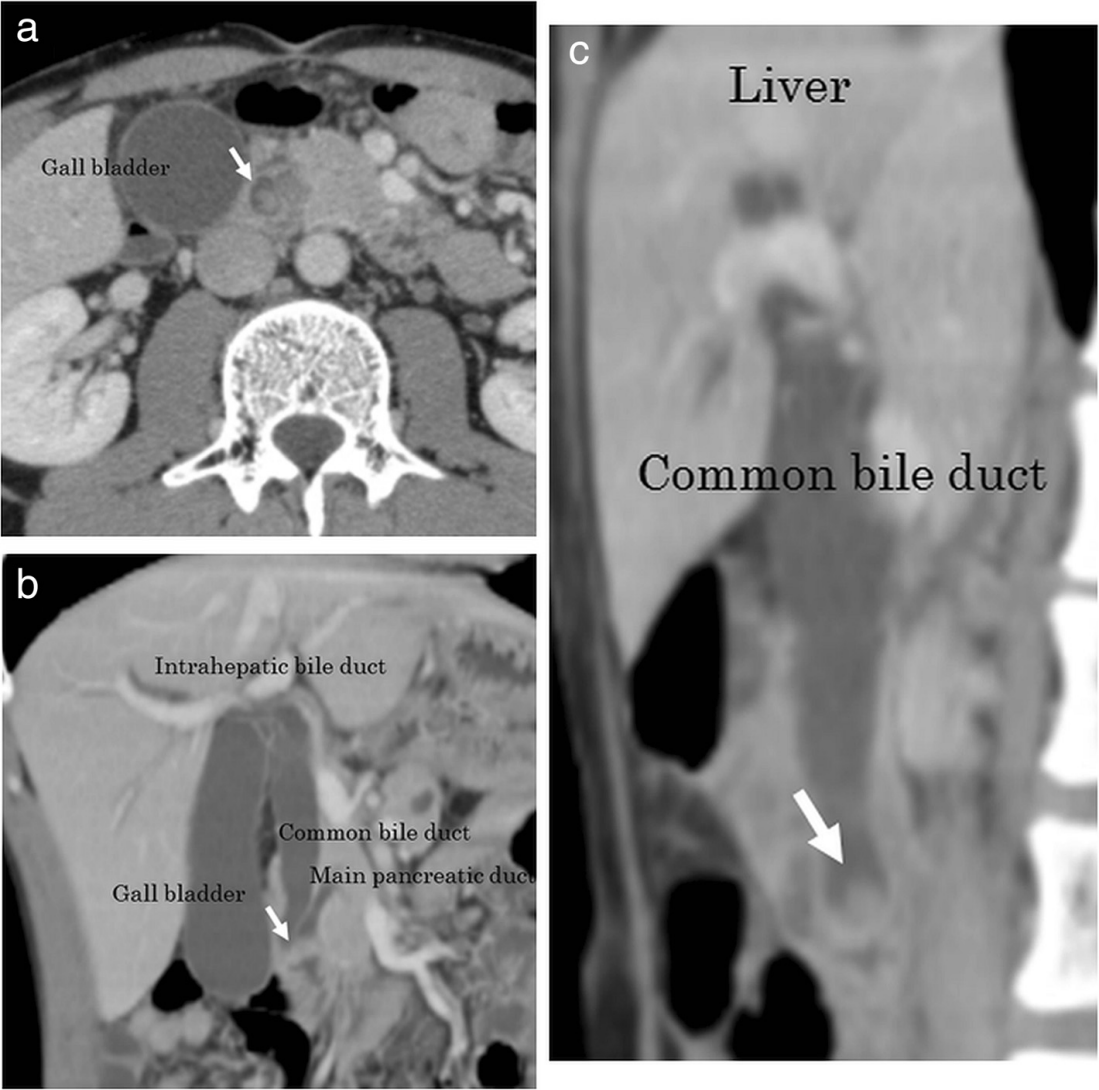
Enhanced CT image showed a hypervascular tumor, 10 mm in diameter, at the papilla of Vater (*arrow*) (**a**) and dilation of the common and intrahepatic bile ducts (*arrow*) (**b**, **c**)

Enhanced computed tomography (CT) showed a hypervascular tumor, 10 mm in diameter, at the papilla of Vater and dilation of the common bile duct (CBD) and the intrahepatic bile duct (Fig. 2a–c), and therefore, detailed studies were performed. Results of laboratory blood tests showed high values of aspartate aminotransferase (169 IU/l), alanine aminotransferase (164 IU/l), alkaline phosphatase (755 IU/l), γ-glutamyltransferase (452 IU/l), total bilirubin (1.96 mg/dl), and pancreatic amylase (382 IU/l).

**Fig. 3:**
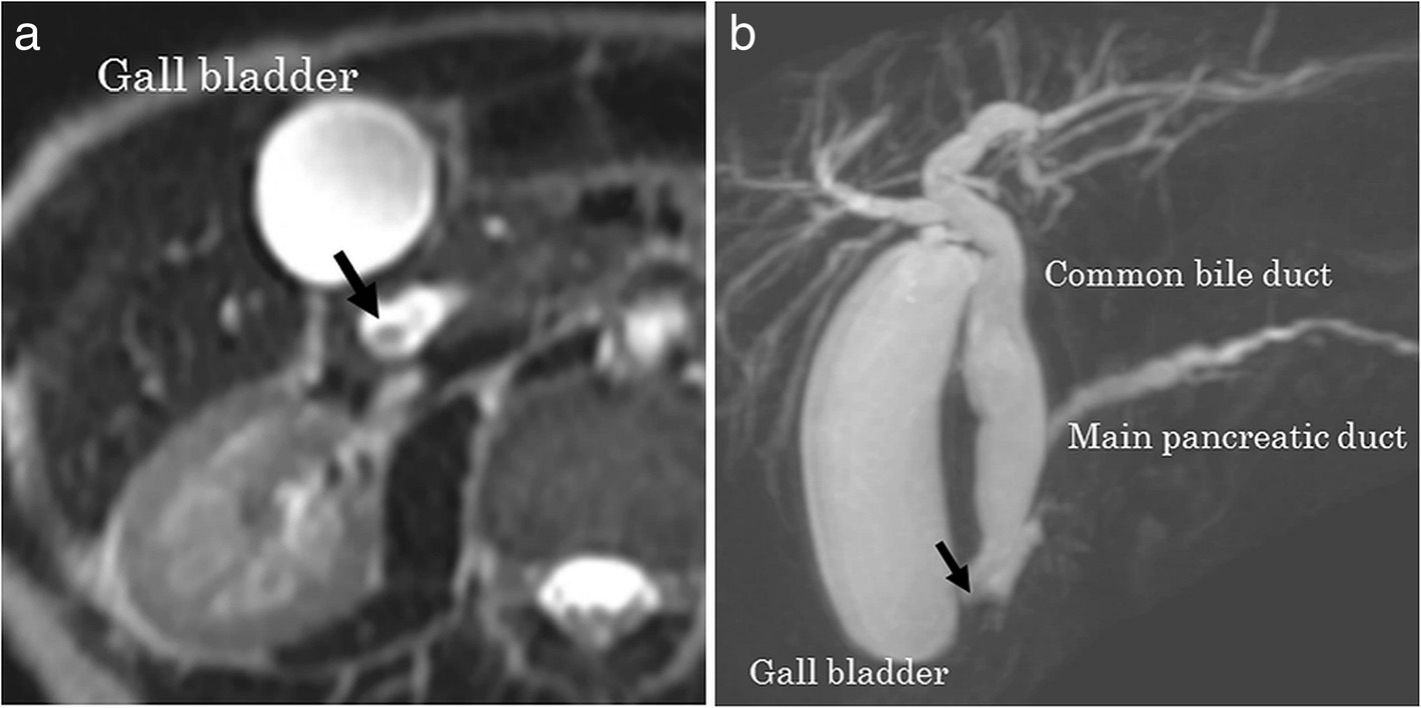
An MRI and MRCP showed the tumor as a defect from the papilla of Vater to the lower CBD (*arrow*) (**a**, **b**)

**Fig. 4:**
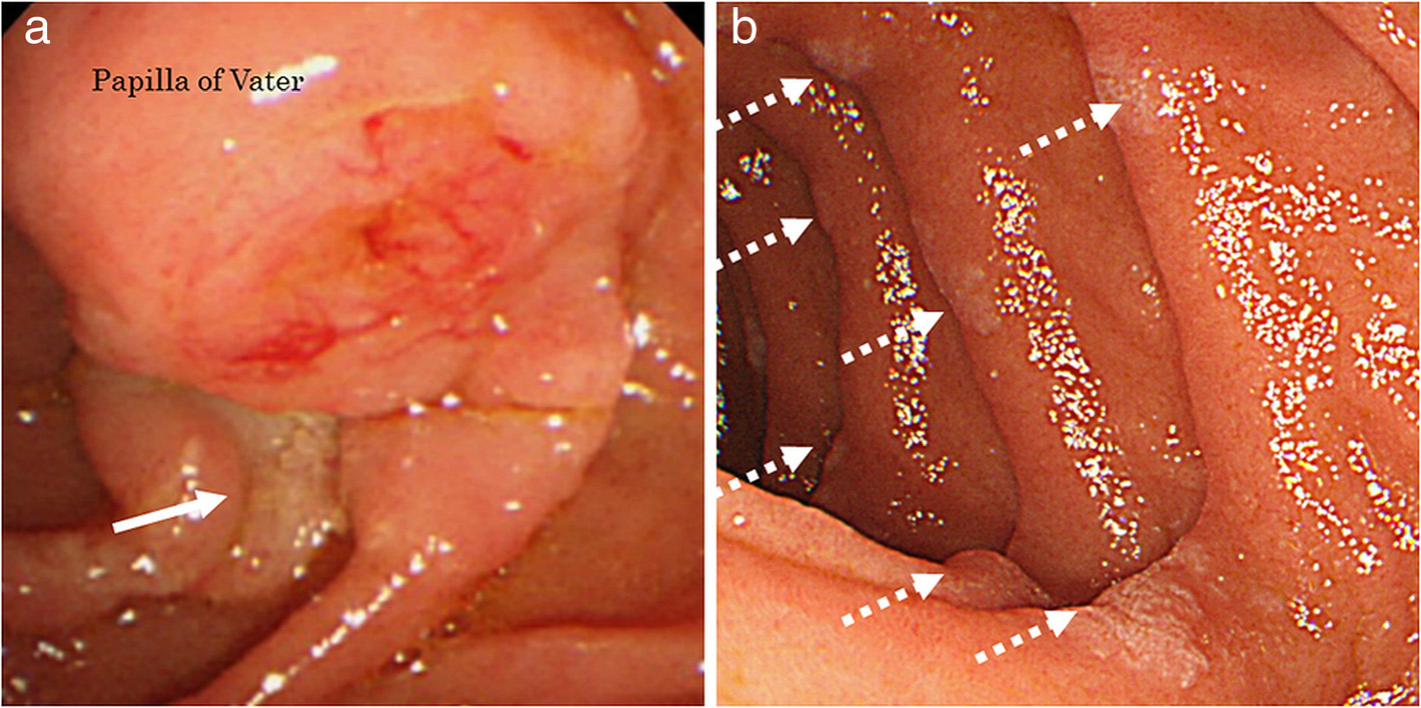
Gastroduodenoscopy revealed a duodenal ulcer nearby the papilla of Vater (*arrow*), which was suspected as the site of tumor invasion (**a**), and a large number of duodenal polyps (*dotted arrows*) (**b**)

**Fig. 5:**
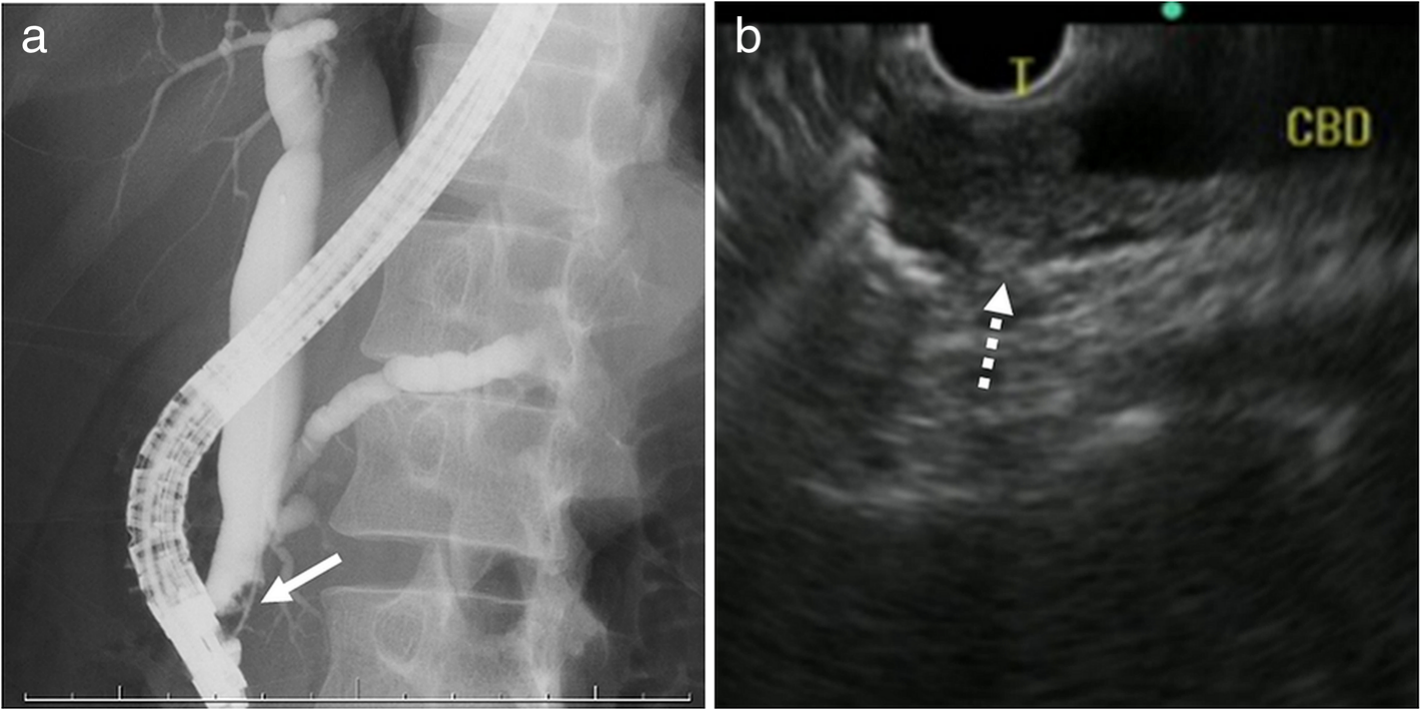
Endoscopic retrograde cholangiopancreatography detected findings similar to those of the MRI and MRCP (*arrow*) (**a**), and IDUS detected a torose lesion (*dotted arrow*) at the lower CBD (**b**)

Magnetic resonance imaging (MRI) and magnetic resonance cholangiopancreatography (MRCP) showed the tumor as a defect present from the papilla of Vater to the lower CBD (Fig. 3a, b). Gastroduodenoscopy showed a large number of gastric and duodenal polyps and a duodenal ulcer nearby the papilla of Vater, which were suspected to be tumor invasion (Fig. 4a, b). Moreover, endoscopic retrograde cholangiopancreatography detected the same findings as those of the MRI and MRCP, and intraductal ultrasonography (IDUS) detected a torose lesion at the lower CBD (Fig. 5a, b). Although a biopsy of the lesion did not reveal any apparent cancer cells, we suspected the tumor to be a malignant tumor of the papilla of Vater because of the hypervascularity shown by CT. No regional lymph node swelling, metastases, or direct invasion to adjacent organs was detected on the chest and abdominal CT images, and there were no other FAP-related findings in other organs. PpPD with modified Imanaga reconstruction, which consists of end-to-side duodenojejunostomy, end-to-side pancreatojejunostomy and choledochojejunostomy, and regional lymph node dissection (D2), were performed.

**Fig. 6:**
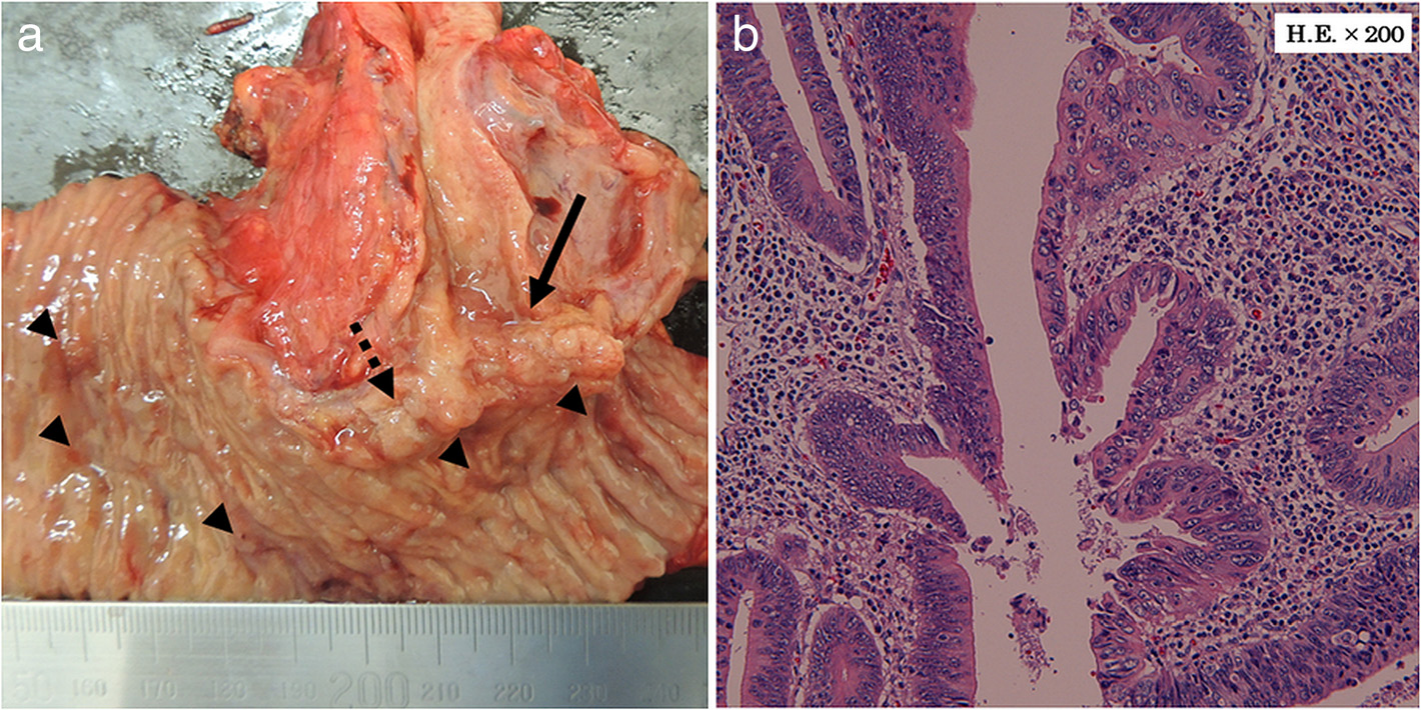
The resected specimen showed a 10-mm circular infiltrating papillary tumor (*arrow*) with a shallow ulcer (*dotted arrow*) at the papilla of Vater and polyps (*arrowheads*) in the duodenal second portion (**a**). The pathological study showed that the tumor was a papillary and moderately differentiated tubular adenocarcinoma (**b**)

The specimen obtained showed a circular infiltrating papillary tumor 10 mm in diameter with a shallow ulcer at the papilla of Vater and a large number of polyps in the duodenal second portion (Fig. 6a). The pathological study indicated that the tumor was a papillary and moderately differentiated tubular adenocarcinoma (pStage: II, invasion level: duodenum alone (T2), lymphatic duct invasion: mild, vessel invasion: mild, neural invasion: not detected) (Fig. 6b). Although a large number of adenomas were found in the portion of the duodenum, regional lymph node, liver, and distant metastasis and peritoneal dissemination were not noted (pN0, H0, M0, P0). The patient is currently in good health without recurrence, weight loss, or severe diarrhea at 12 months after surgery.

### Discussion

Cancer of the duodenum and the papilla of Vater affects the prognosis of patients with FAP. Many of these patients have adenomatous polyps in the second and third portions of the duodenum, and although the polyps remain adenomatous in many cases, 5 % of the polyps change from adenoma to carcinoma via the adenoma-carcinoma sequence [2, 4, 8, 10]. Interestingly, Kadmon et al. reported that no clear association between the number of colon polyps and the number of upper gastrointestinal polyps has been identified [11], whereas the presence of colorectal cancer is related to the increase in duodenal or periampullary cancer in patients with FAP [12]. Therefore, patients with FAP independently need to undergo periodic gastroduodenoscopy and side-viewing endoscopy such as colonoscopy.

Polyps in the periampullary area can cause obstruction of the biliary and pancreatic ducts, resulting in elevation of bilirubin and hepatobiliary pancreatic enzymes, jaundice, or pancreatitis as in our case [10], which suggests that awareness of biliary-pancreatic symptoms yields an opportunity to check for periampullary polyps in patients with FAP along with periodic gastroduodenoscopy. However, this does not diminish the primary importance of periodic medical surveillance for FAP patients.

According to the Spigelman scoring system, several treatments, among which are endoscopic mucosal resection, endoscopic ampullectomy, and prophylactic PpD/PpPD, are applicable to stage IV patients; however more invasive treatments, such as surgical procedures including PpPD, are applicable to cancer patients [5–8]. The risk of recurrence of both stage IV disease and cancer remains substantial. Especially, less invasive treatments result in high rates of recurrence of adenomas/cancer: rates following ampullectomy for early ampullary cancer range from 10 to 12.8 %, and that following PpD for duodenal adenomas/cancer is 50 % [7, 12–14]. Therefore, individual patient characteristics need to be carefully considered when adopting a less invasive treatment.

For duodenal or periampullary cancer, there would be no objection to performing pancreaticoduodenectomy (PD), and thus in many cases, PpPD is performed. Several authors have reported no difference in perioperative morbidity, long-term survival, and other factors between PD and PpPD. However, except for the incidence of delayed gastric emptying, PpPD is better than PD in terms of operating time, blood loss, nutritional status, and capacity to work at 6 months after surgery [15–19]. The Whipple or child reconstruction procedure is usually performed to avoid complications of pancreatic fistula (PF), abdominal abscess, or hemorrhage. However, these procedures do not create a physiological route; the modified Imanaga reconstruction is a physiological procedure that offers good long-term nutritional status and allows easier postoperative observation and treatment of the pancreatic/biliary duct [20]. The weak point of the modified Imanaga reconstruction is that it occasionally intensifies the severity of PF or cholangitis more than that by the Whipple or child procedure, and it delays ingestion when PF occurs. Nevertheless, there are at least two benefits of the modified Imanaga reconstruction in FAP: ease of endoscopic intestinal observation/treatment and maintenance of nutritional status after subtotal colectomy for colorectal lesions of FAP. Therefore, we selected the modified Imanaga procedure for our patient. However, after performing the PpPD in our patient, we experienced a patient with duodenal cancer arising from the remaining duodenum after PpPD for ampullary cancer in FAP [21], suggesting that resection of the pylorus and the duodenal bulb with pylorus-resecting PD (PrPD) can remove all of the duodenum and contributes to the prevention of any remaining duodenal cancer in patients with FAP. Moreover, long-term outcomes of PrPD are similar to those of PpPD, and PrPD reduces the incidence of delayed gastric emptying compared with PpPD [22–24]. Thus, PrPD with the modified Imanaga procedure might be a better option in patients with FAP. Further evaluation with high-quality prospective studies is necessary.

## Conclusions

In the future, expanded oncological, pathological, and surgical knowledge of duodenal or periampullary polyps and cancer associated with FAP might bring many FAP patients more relevant treatment than they receive at present. Although more study is necessary, PrPD with the modified Imanaga reconstruction has the potential to become one of the more effective procedures for the treatment of FAP.

## Consent

Written informed consent was obtained from the patient for publication of this case report and accompanying images. A copy of the written consent is available for review by the Editor-in-Chief of this journal on request.

## Abbreviations

CBD: common bile duct
CT: computed tomography
FAP: familial adenomatous polyposis
IDUS: intraductal ultrasonography
MRCP: magnetic resonance cholangiopancreatography
MRI: magnetic resonance imaging
PD: pancreaticoduodenectomy
PF: pancreatic fistula
PpD: pancreas-preserving duodenectomy
PpPD: pylorus-preserving pancreaticoduodenectomy
PrPD: pylorus-resecting pancreaticoduodenectomy
